# Mitochondrial Common Deletion, a Potential Biomarker for Cancer Occurrence, Is Selected against in Cancer Background: A Meta-Analysis of 38 Studies

**DOI:** 10.1371/journal.pone.0067953

**Published:** 2013-07-04

**Authors:** Hezhongrong Nie, Hongying Shu, Rasika Vartak, Amanda Claire Milstein, Yalin Mo, Xiaoqin Hu, Hezhi Fang, Lijun Shen, Zhinan Ding, Jianxin Lu, Yidong Bai

**Affiliations:** 1 Zhejiang Provincial Key Laboratory of Medical Genetics, School of Life Sciences, Wenzhou Medical College, Wenzhou, Zhejiang, PR China; 2 Department of Cellular and Structural Biology, University of Texas Health Sciences Center at San Antonio, San Antonio, Texas, United States of America; University of Medicine and Dentistry of New Jersey, United States of America

## Abstract

Mitochondrial dysfunction has been long proposed to play a major role in tumorigenesis. Mitochondrial DNA (mtDNA) mutations, especially the mtDNA 4,977 bp deletion has been found in patients of various types of cancer. In order to comprehend the mtDNA 4,977 bp deletion status in various cancer types, we performed a meta-analysis composed of 33 publications, in which a total of 1613 cancer cases, 1516 adjacent normals and 638 healthy controls were included. When all studies were pooled, we found that cancerous tissue carried a lower mtDNA 4,977 bp deletion frequency than adjacent non-cancerous tissue (OR = 0.43, 95% CI = 0.20–0.92, *P* = 0.03 for heterogeneity test, *I^2^* = 91.5%) among various types of cancer. In the stratified analysis by cancer type the deletion frequency was even lower in tumor tissue than in adjacent normal tissue of breast cancer (OR = 0.19, 95% CI = 0.06–0.61, *P* = 0.005 for heterogeneity test, *I^2^* = 82.7%). Interestingly, this observation became more significant in the stratified studies with larger sample sizes (OR = 0.70, 95% CI = 0.58–0.86, *P* = 0.0005 for heterogeneity test, *I^2^* = 95.1%). Furthermore, when compared with the normal tissue from the matched healthy controls, increased deletion frequencies were observed in both adjacent non-cancerous tissue (OR = 3.02, 95% CI = 2.13–4.28, *P*<0.00001 for heterogeneity test, *I^2^* = 53.7%), and cancerous tissue (OR = 1.36, 95% CI = 1.04–1.77, *P* = 0.02 for heterogeneity test, *I^2^* = 83.5%). This meta-analysis suggests that the mtDNA 4,977 bp deletion is often found in cancerous tissue and thus has the potential to be a biomarker for cancer occurrence in the tissue, but at the same time being selected against in various types of carcinoma tissues. Larger and better-designed studies are still warranted to confirm these findings.

## Introduction

Mitochondria are ubiquitous organelles in eukaryotic cells with the primary function to generate energy in the form of ATP through the coupling of oxidative phosphorylation (OXPHOS) [1]. Mitochondria contain their own genome. In human, mitochondrial genome is a 16.5 kb compactly organized, double-stranded, and closed molecule [2]. Human mtDNA contains 37 genes encoding 13 polypeptides, all of which are the components of the respiratory chain/OXPHOS system, 2 ribosomal RNAs and 22 transfer RNAs [2], [3]. Due to the lack of a sophisticated DNA repair mechanism, mtDNA is more prone to attacks by reactive oxygen species (ROS), a byproduct of respiration, and the somatic mutation rate of mtDNA is presumed to be 10 to 20 times higher than that of nuclear DNA [4], [5].

Defects in mitochondrial function have long been hypothesized to play a role in tumorigenesis [6], and a large number of mtDNA mutations have been detected in a variety of cancers [7], [8]. Reported sequence changes in cancer patients including point mutations, multiple deletions and microsatellite instability (MSI) in coding and noncoding regions [9]–[12]. One of the best-described mtDNA mutation is the mtDNA 4,977 bp or common deletion [13], which deletes between nucleotides 8,470 and 13,447 of the human mtDNA. This mutation removes all or part of the genes encoding four complex I subunits, one complex IV subunit, two complex V subunits and five tRNA genes, which are indispensable for maintaining normal mitochondrial function [14].

The mitochondrial common deletion has attracted tremendous interests as it is associate with several sporadic diseases including myopathies, Alzheimer disease, Pearson’ s syndrome, photoaging of the skin, Kearns-Sayre syndrome (KSS) and chronic progressive external ophthalmoplegia (CPEO) [5], [13], [15]–[19]. Furthermore, this deletion also accumulates in many tissues during aging, and has been used as an indication of mtDNA oxidative damage [20], [21]. Although many studies associate this common deletion with tumorigenesis, results from population studies remain conflicting rather than conclusive [22]–[24]. Several studies have found the mtDNA 4,977 bp deletion in various types of cancer, including in cancer of the breast, endometrium, esophagus, stomach, head and neck, liver, lung, oral, kidney, skin and thyroid [22], [23]. In some cases, the incidence and level of the 4,977 bp deletion were lower in the cancer tissue compared with adjacent non-cancerous tissue from the same patients [24]. In some reports no significant associations were detected [25]. Mitochondrial DNA deletions serve as biomarkers of aging in the skin, but are typically absent in non melanoma skin cancers. Thus, the role of this common deletion in tumorigenesis is intriguing, but largely perplexing, and each of these single studies may have been underpowered to detect the association between mtDNA common deletion and cancers because of their small sample size. In the hope that the underlying heterogeneity among different studies can be resolved in a large scale analysis, we conducted a systematic meta-analysis on 33 relevant published articles with 1613 cases, 1516 adjacent normals and 638 healthy controls to drive a more precise estimation, and with an improved statistic power to detect the association of this mtDNA alteration with various cancer types.

## Materials and Methods

### Search Strategy and Selection Criteria

We carried out a search in Pubmed and other public domains with a combination of the following keywords: ‘4,977 bp deletion’, ‘cancer’ or ‘carcinoma’, and ‘risk’ to identify all case-control studies published to date on an association between mitochondrial DNA 4,977 bp deletion and various types of cancer (last search update in November, 2012). Additional studies were identified by a hands-on search of bibliographies of original studies on this topic.

Studies were included if they meet the following criteria: full-text articles in English; case-control design study; odds ratios (ORs) in case-control studies were reported with the 95% confidence intervals (CIs) (or, if 95% CIs were not reported, the reported data were sufficient to calculate them); if more than one article was published using the same patient population, only the latest or the complete study would be used in this analysis. We excluded studies if the crucial data were not reported in original papers, or if they had a very high probability of inaccurate reporting.

### Data Extraction

Data were independently extracted by two authors (Hezhongrong Nie and Hongying Shu) and checked by the other author, any disagreement was resolved by discussions until consensus was reached. The following information was extracted from all included publications: the first author, year of publication, cancer types, total number of cases and controls, country and ethnicity of the study population, DNA source, number of included patients, deletion detection method and deletion results of each study. For studies including subjects of different racial descents, data were extracted separately for each ethnic group categorized as European ancestry (EA), Asian or others. When a study did not state the ethnic group type or if it was impossible to separate participants according to the data presented, the sample was termed as ‘others’. Furthermore, references involving different cancer type, different ethnic group, different sample size, different DNA source and different measurement method were divided into multiple study samples for subgroup analyses.

### Quantitative Data Synthesis

The numbers of cases and controls by deletion status from each study were collected to evaluate this deletion frequency among various types of cancer (ORs and 95% CIs), meanwhile, the median of the relative deletion level were also collected from some studies who displayed the data (data not shown). Furthermore the stratification analyses were also conducted by ethnicity (EA or Asian), cancer type (if one cancer type was investigated in less than two studies, it would be merged into the ‘other cancers’ group), sample size (not larger than 50 and larger than 50), measurement method and DNA source.

### Statistical Analysis

Heterogeneity was quantified with the *X* ^2^-based Q test and *I^2^* statistic, using Q test to assess between-study heterogeneity and considered significant if *P*<0.05 [26], and *I^2^* statistic can work out a value that indicates what proportion of the total variation across studies is beyond chance. Specifically, 0% indicates no observed heterogeneity, and larger values show increasing heterogeneity [27]. The choice of fixed-effects model or the random-effects model was based on the Mantel-Haenszel method and the DerSimonian and Laird method. When *P* value of the heterogeneity test was≥0.05, the fixed-effects model was used, which assumes the same homogeneity of effect size across all studies [28]. Otherwise, the random-effects model was more appropriate, which tends to provide wider confidence intervals as the results of the constituent studies differ among themselves [29]. Subgroup analyses were also performed by ethnic group, cancer type, sample size, measurement method and DNA source. To assess the effects of individual studies, sensitivity analysis was performed by excluding each study at a time individually and recalculating the ORs and confidence intervals. Potential publication bias was estimated by the inverted funnel plot, in which the standard error of log (OR) of each study was plotted against its log (OR) [30], and an asymmetric plot suggests a possible publication bias. Funnel plot asymmetry was assessed by Begg’s and Egger’s linear regression test. The significance of the intercept was determined by the *t* test as suggested by Egger, and *P*<0.05 was considered representative of statistically significant publication bias [30]. This meta-analysis was performed by using the software Review Manage (v.4.2) and Stata 12.0 (Stata Corporation, College Station, TX). All the *P*-values were two-sided, and a *P*<0.05 was considered statistically significant.

## Results

### Study Characteristics

A total of 768 potentially relevant records were identified by using the key words mentioned earlier in the Methods, of which 50 were examined the mtDNA 4,977 bp deletion frequency in cancers after the title and abstract review. After full-text review, 17 were excluded because of several reasons such as they did not provide available data or the studies were reviews. Finally, a total of 38 studies in 33 articles met our inclusion criteria. The characteristics of each case-control study are listed in **Table 1**. Of the 38 studies, sample sizes ranged from 7 to 130, in which nine studies focusing on breast cancer [25], [31]–[38], five studies focusing on hepatocellular carcinoma [39]–[43], four focusing on gastric cancer [38], [44]–[46], three focusing on colorectal cancer [25], [38], [47], three focusing on esophageal cancer [48]–[50], three focusing on oral cancer [51]–[53], two focusing on thyroid cancer [25], [54], two focusing on skin cancer [55], [56] and seven studies of other cancers (one endometrial carcinoma [57], one lung cancer [58], one Warthin’s cancer [59], one cervix cancer [60], one acute lymphoblastic leukemia [61], one prostate cancer [62] and one head and neck cancer [38]). Because some controls in two researches [25], [38] were shared by several cancers, it was defined as four studies (breast cancer, colorectal cancer, gastric cancer and head and neck cancer) and three studies (thyroid cancer, breast cancer and colorectal cancer) in the analysis stratified by cancer type but defined as one study in the overall analysis and stratification analysis by ethnicity, sample size, measurement method and DNA source. Overall, 12 studies used EA, 20 used Asians and one used other ethnic groups. These were 27 case/adjacent normal studies and 15 case/healthy normal studies included. Additionally, the tissue was the most common source of DNA, although other sources were also applied, such as blood and buccal swab [25], [32], [38], [48], [49]. Buccal swab was termed as tissue in the stratified analysis by DNA source.

**Table 1:** Characteristics of studies included in the meta-analysis.

				Group 1^a^	Group 2^b^	Group 3^c^		
Surname, year	Country	Ethnicity	Cancer type	(n^d^/N^e^)	(n/N)	(n/N)	DNA source	Measurement method
Kamalidehghan, 2006	Iran	Asian	gastric	6/107	18/107		tissue	regular PCR
Tan, 2009	UK	EA	esophageal	2/12	9/10	1/12	tissue	regular PCR
Bianchi, 1995	Argentina	EA	breast	1/7	3/7		tissue	regular PCR
Aral, 2010	Turkey	EA	thyroid, breast and colorectal	4/100	3/100	0/49	tissueand blood	regular PCR
Chen, 2011	China	Asian	colorectal	17/104	13/104		tissue	regular PCR
Abnet, 2004	China	Asian	esophageal	17/19	19/20		tissue and blood	regular PCR
Dai, 2006	China	Asian	lung	20/37	22/37	6/20	tissue	regular PCR
Dani, 2004	Brazil	others	breast, colorectal, gastric and head and neck	43/87	74/87	2/17	tissue and blood	regular PCR
Gwak, 2011	Italy	EA	hepatocellular	3/27	24/27	8/8	tissue	regular PCR
Kamenisch, 2007	Germany	EA	skin	37/41	40/41		tissue	regular PCR
Kara, 2012	Turkey	EA	cervix	4/21		5/16	tissue	regular PCR
Futyma, 2008	Poland	EA	endometrial	30/37	32/37		tissue	regular PCR
Lee, 2001	Taiwan	Asian	oral	26/53	36/53		tissue	regular PCR
Lewis, 2000	UK	EA	Warthin’s tumor	14/14		6/6	tissue	qPCR^f^
Rahul, 2012	India	Asian	oral	42/50		18/50	tissue	regular PCR
Upadhyay, 2009	India	Asian	esophageal	2/39	1/39		tissue	regular PCR
Shao, 2004	China	Asian	hepatocellular	19/27	12/27		tissue	qPCR
Fukushima, 1994	Japan	Asian	hepatocellular	7/28		23/35	tissue	regular PCR
Tan, 2003	Taiwan	Asian	oral	2/18	5/18		tissue	regular PCR
Tan, 2012	USA	EA	breast	0/19	0/19		tissue	regular PCR
Tseng, 2006	Taiwan	Asian	breast	3/60	28/60		tissue	regular PCR
Tseng, 2009	Taiwan	Asian	breast	3/60	29/60		tissue	regular PCR
Maximo, 2002	Porto	EA	thyroid	26/44	9/42		tissue	regular PCR
Pavicic, 2009	Argentina	EA	breast	43/95	70/95	78/199	tissue	regular PCR
Wang, 2009	China	Asian	gastric	86/108	73/108	29/56	tissue	regular PCR
Wen, 2011	China	Asian	ALL^g^	26/26		39/39	blood	qPCR
Wheelhouse, 2005	China	Asian	hepatocellular	17/62	59/62	9/9	tissue	regular PCR
Wu, 2005	Taiwan	Asian	gastric	3/31	17/31		tissue	regular PCR
Yang, 2004	Taiwan	Asian	skin	12/17		26/53	tissue	regular PCR
Ye, 2008	China	Asian	breast	76/76		76/76	tissue	qPCR
Yin, 2004	Taiwan	Asian	hepatocellular	18/18	18/18		tissue	regular PCR
Yu, 2010	China	Asian	prostate	98/130	14/130		tissue	regular PCR
Zhu, 2004	USA	EA	breast	18/39	13/39	6/23	tissue	regular PCR

a cancerous tissue.

b non-cancerous tissue.

c healthy normal tissue.

d deletion number.

e total number.

f quantitative PCR.

g acute lymphoblastic leukemia.

### Meta Analysis Results

**Figure 1:**
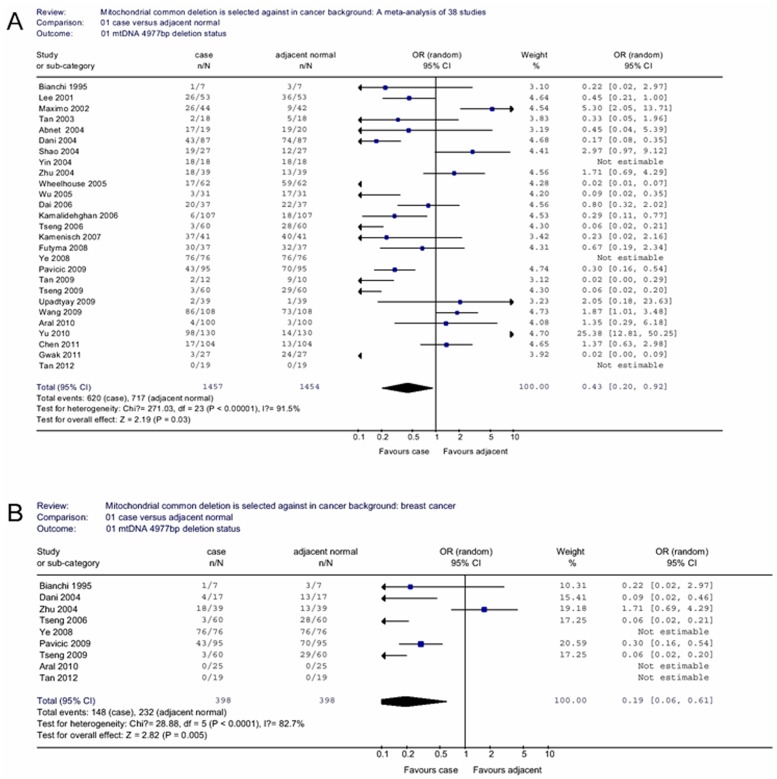
Odds ratios (ORs) and 95% confidence intervals (CIs). (A) various cancer types associated with mtDNA 4977 bp deletion in case/adjacent normal group. (B) stratified analysis for breast cancer associated with mtDNA 4977 bp deletion in case/adjacent normal group.

We obtained the mtDNA 4977 bp deletion data from 33 publications consisting of 1613 cases, 1516 adjacent normal and 638 healthy controls. When all eligible studies were pooled into the meta-analysis, we found that cancerous tissue carried a lower mtDNA 4,977 bp deletion frequency than adjacent non-cancerous tissue (OR = 0.43, 95% CI = 0.20–0.92, *P* = 0.03 for heterogeneity test, *I^2^* = 91.5%; **Fig. 1A**). However, no significant association was found if the samples were divided into EA subject group (OR = 0.43, 95% CI = 0.11–1.73, *P* = 0.23 for heterogeneity test, *I^2^* = 91.4%) or Asian subject group (OR = 0.49, 95% CI = 0.17–1.44, *P* = 0.20 for heterogeneity test, *I^2^* = 93.3%). In the stratified analysis by cancer type (**Table 2**), we found that the difference of detection of 4,977 bp deletion in cancerous samples and corresponding non-cancerous breast samples was more pronounced (OR = 0.19, 95% CI = 0.06–0.61, *P* = 0.005 for heterogeneity test, *I^2^* = 82.7%; **Fig. 1B**); but this phenomenon did not exist in hepatocellular cancer (OR = 0.10, 95% CI = 0.00–3.74, *P* = 0.21 for heterogeneity test, *I^2^* = 95.4%), gastric cancer (OR = 0.33, 95% CI = 0.06–1.67, *P* = 0.18 for heterogeneity test, *I^2^* = 86.9%), esophageal cancer (OR = 0.28, 95% CI = 0.02–3.73, *P* = 0.34 for heterogeneity test, *I^2^* = 69.0%) and colorectal cancer (OR = 0.66, 95% CI = 0.37–1.16, *P* = 0.15 for heterogeneity test, *I^2^* = 87.6%).

**Table 2:** The mtDNA 4977 bp deletion status in stratification analysis by selected factors.

	No.of			No.of		
Stratification	studies^a^	OR(95%CI)^a^	*P*_het_ a,b	studies^c^	OR(95%CI)^c^	*P*_het_ b,c
**Ethnicity**						
Caucasian	10	0.40 (0.13–1.25)	0.11	7	0.97 (0.34–2.78)	0.95
Asian	16	0.49 (0.17–1.44)	0.2	6	0.96 (0.16–5.66)	0.97
**Cancer type**						
Breast cancer	9	0.19 (0.06–0.61)	0.005	4	0.44 (0.28–0.70)	0.0005
Gastric cancer	4	0.33 (0.06–1.67)	0.18	1	–	–
Colorectal cancer	3^d^	–	–	–	–	–
Esophageal cancer	3	0.28 (0.02–3.73)	0.34	–	–	–
Hepatocellular cancer	4	0.10 (0.00–3.74)	0.21	3^e^	–	–
**Sample size**						
≤50	15	0.46 (0.18–1.19)	0.11	10	1.09 (0.32–3.64)	0.89
>50	12	0.70 (0.58–0.86)	0.0005	5	1.60 (1.13–2.27)	0.009
**Method**						
Regular PCR	25	0.39 (0.18–0.86)	0.02	13	1.36 (0.62–2.97)	0.44
qPCR	2	–	–	2	–	–

a Case/adjacent normal group.

b *P* value of the Q-test for heterogeneity test.

c Case/healthy normal group..

d One study was excluded..

e Couldn’t define its heterogeneity.

**Figure 2:**
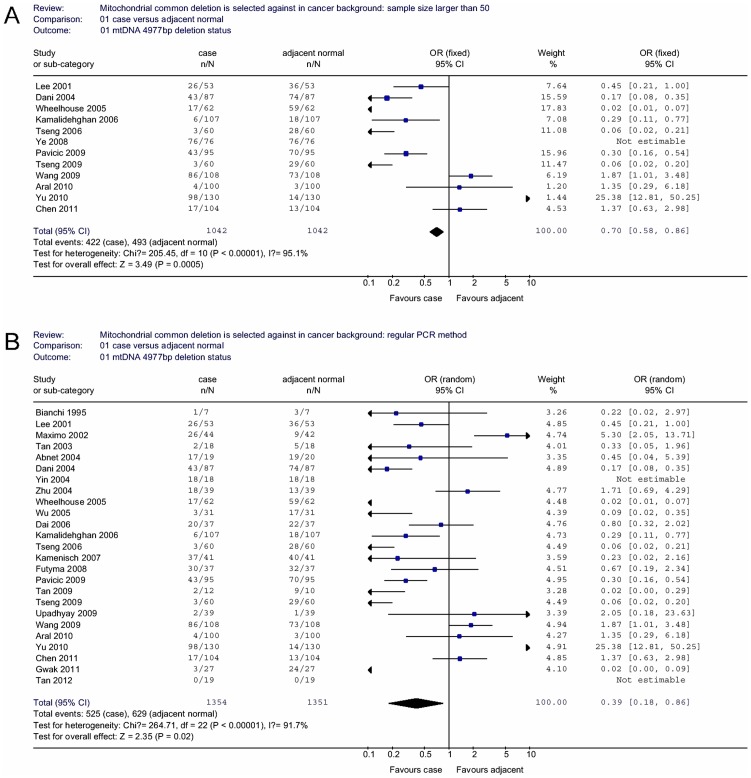
Odds ratios (ORs) and 95% confidence intervals (CIs) for various cancer types associated with mtDNA 4977 bp deletion in case/adjacent normal group. (A) sample size larger than 50. (B) regular PCR measurement method.

As breast cancer has been the most studied one with the implication of mitochondrial dysfunction and mtDNA in tumorigenesis, we examined the role of sample size in the detection of the effect of common deletion. Interestingly, we found the difference in deletion frequency in cancer tissue and in adjacent non-cancerous tissue was becoming more significant when the sample size is larger than 50 (OR = 0.70, 95% CI = 0.58–0.86, *P* = 0.0005 for heterogeneity test, *I^2^* = 95.1%; **Fig. 2A**). As for the experimental methods, the regular PCR appeared to be more sensitive (OR = 0.39, 95% CI = 0.18–0.86, *P* = 0.02 for heterogeneity test, *I^2^* = 91.7%; **Fig. 2B**).

**Figure 3:**
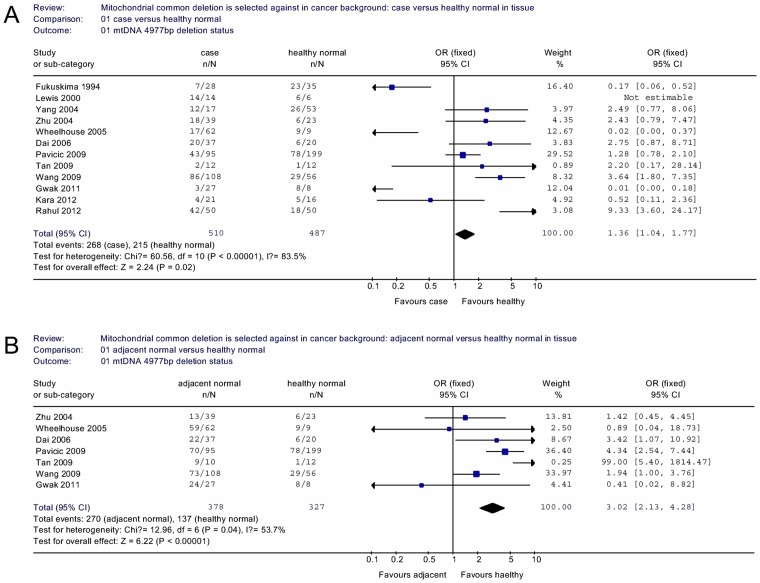
Odds ratios (ORs) and 95% confidence intervals (CIs) for various cancer types associated with mtDNA 4977 bp deletion. (A) case/healthy normal group. (B) adjacent normal/healthy normal group of tissue sample stratified analysis.

To further determine if mtDNA common deletion could serve as a potential marker for tumorigenesis, we then compared the frequencies of common deletion detection in cancer patient, cancerous tissue and adjacent non-cancerous tissues with tissues from the healthy controls, we found it was more likely to detect mtDNA common in tissues from the cancer patients in both cancerous (OR = 1.36, 95% CI = 1.04–1.77, *P* = 0.02 for heterogeneity test, *I^2^* = 83.5%; **Fig. 3A**) and adjacent non-cancerous (OR = 3.02, 95% CI = 2.13–4.28, *P*<0.00001 for heterogeneity test, *I^2^* = 53.7%; **Fig. 3B**) tissues.

### Sensitivity Analyses

We performed sensitivity analysis to assess the stability of the results of this meta-analysis by sequentially excluding each study in both case/adjacent normal and case/healthy normal groups. The leave-one-out sensitivity analysis indicated that no single study changed the pooled ORs qualitatively, suggesting the stability of this meta-analysis (**Fig. S1A and Fig. S1B**).

### Publication Bias

Publication bias was assessed by Begg’s funnel plot and Egger’s test. As shown in **Fig. S2A** and **Fig. S2B**, the shapes of the funnel plots seemed symmetrical in both case/adjacent normal and case/healthy normal groups, suggesting the absence of publication bias. Meanwhile, the Egger’s test was performed to provide statistical evidence of funnel plot asymmetry. The results indicated no significant evidence for publication bias of the current meta-analysis (*P* = 0.081 in case/adjacent normal group and *P* = 0.573 in case/healthy normal group). All together, we believe that bias from publications might not have a significant influence on the results of our meta-analysis.

## Discussion

In this meta-analysis of 1613 cancer cases, 1516 adjacent normals and 638 healthy controls from 33 independent publications, while the mtDNA 4,977 bp deletion was found in both cancerous and adjacent normal tissue, when compared to matched healthy controls, the deletion frequency was significantly lower in tumor tissue than in adjacent non-cancerous tissue among various types of cancer. This indicates that the mtDNA 4,997 bp deletion is selected against in cancerous tissue as compared to the adjacent normal tissue, making it an attractive biomarker for cancer occurrence.

Mitochondrial DNA is continuously exposed to oxidative stress, thus accumulating a large load of mutations, one of the most common being the mtDNA 4,977 bp deletion [22]. The mtDNA 4,977 bp deletion has been reported in several kinds of cancers, such as breast cancer [31], [34], [35], [38], colorectal cancer [38], gastric cancer [38], [45], [46], hepatocellular carcinoma [42], [43], lung cancer [58], head and neck cancer [38], esophageal cancer [63] and thyroid carcinoma [64]. Also not surprisingly, due to the low specificity of this common deletion, mtDNA 4,977 bp deletion has also been shown to be implicated in the occurrence of various types of degenerative diseases and aging [43], [65], [66] as well as in healthy infants and children [67]. Both animal studies and cell model analysis showed the mtDNA 4,977 bp deletion played an important role during the course of tumorigenesis [68], [69]. However, the results of various studies dissecting the role of this mutation in cancer development are conflicting. For example, the study of Ye found that though all of their samples carry mtDNA 4,977 bp large deletion, they thought there was no correlation between mtDNA 4,977 bp common deletion and cancer risk [34]; Tseng et al demonstrated that the detection frequency of common deletion was more higher in adjacent normal tissue than carcinoma tissue in their 60 breast cancer patients from Taiwan [35]; meanwhile, the analysis of Dani concurred with the results from Tseng in several cancer types [38]. The mtDNA 4,977 bp common deletion mutations is a highly non-specific mutation and as mentioned before has been seen in degenerative disorders as well as in healthy tissues. This can account for the inconsistency in the data obtained so far. Clearly, other cellular as well as tissue specific environmental factors may also affect the outcome of this mutation on the cell survival and tumorigenesis. As for the proportion of this deletion in cancer tissues, it may be influenced by many factors, including its presence in cancer precursor cell(s) and the growth rate for cancer cells. Hence, while a promising observation, future studies are required to determine whether the mutation can be used as a successful biomarker for cancer. Our previous studies have shown that heteroplasmic DNA mutation, where the burden of mutation is less, was more tumorigenic than homoplasmic mutation where all mtDNA is mutated [70]. Our studies have also shown a difference in the retrograde signaling pathways between homoplasmic mitochondria and heteoplasmic mitochondria with lesser load of a particular mutation activating survival pathways involving ROS and Akt thus making cells tumorigenic while inhibition of tumorigenesis was seen in cells with homoplasmic mutations. This supports our hypothesis that a larger mutation load may be required during early stages to tumor development, the mutation being then selected against if the cells have to continue the proliferative growth leading to a greater frequency of mutations in adjacent normal tissue as compared to cancerous tissue. The mtDNA 4,977 bp deletion can thus serve as a potential biomarker for early stage tumor development. The inconsistent results may also due to the individual studies with a small sample sizes and thus not enough statistical power to detect the reliable association. Therefore, to deal with the problem of sample size and to understand the role of this mutation in cancer, we conducted this meta-analysis where we found that the mtDNA 4,977 bp deletion were less frequent in various cancer tissues as compared with adjacent non-cancerous tissues.

Although this meta-analysis showed the mtDNA common deletion was more likely happening in different types of adjacent non-cancerous tissues than in cancerous tissues, some results from stratification analysis remind us of drawing the conclusion with caution. The stratified analysis by cancer type showed that the mtDNA 4,977 bp deletion was more likely in adjacent non-cancerous tissue of breast cancer patients (*P* = 0.005), which contains the largest sample size in our cancer type stratified analysis. Our heterogeneity analysis showed that cancer type did contribute to substantial heterogeneity, these inconsistent results by cancer types may involve different carcinogenic mechanisms. One possible explanation for the differences found between different non-cancerous tissue was the existence of tissue-specific mtDNA turnover rates and various environmental and genetic influences. Different biological pathways (such as repair of oxidative damage, metabolism of hormone and age-related cell signal pathway) could interact with mtDNA, resulting in different efforts on mtDNA damage and cancer susceptibility. For example, several studies found the effect of mtDNA 4,977 bp deletion on colorectal cancer was significant for certain subgroups, such as patients under 65 years old were more likely to carry this deletion in tumor tissues (*P* = 0.027) [47]. This result indicated that there is possibly a negative selection for the common deletion in tumor tissues during aging [47].

Furthermore, the significantly lower mtDNA 4,977 bp deletion frequency in cancer tissue compared with adjacent non-cancerous tissue of larger sample size studies (OR = 0.70, 95% CI = 0.58–0.86, *P* = 0.0005 for heterogeneity test, *I^2^* = 95.1%) suggests to us that genetic risk of cancer conferred by the common variants was very modest and the penetrance of the variants was very small, which means that even if the variation was crucial for carcinogenesis, extremely large-scale evidence would be necessary to establish with high confidence the presence of specific associations.

However, the results from stratification analysis by measurement method indicated that the tumor tissue deletion frequency was also lower than the adjacent non-cancerous tissue in studies with the detection method of regular PCR (OR = 0.39, 95% CI = 0.18–0.86, *P* = 0.02 for heterogeneity test, *I^2^* = 91.7%). But we considered the statistical power was limited in the analyses of such subgroups, these studies suffer from several major drawbacks, such as information bias, selection bias, lower study size and inferior statistical power, which may have a substantial influence on the results of our meta-analysis. Considering those limitations included in the stratified analysis, our results should be interpreted with caution since it may not have enough statistic power. In measurement method stratification analysis, only two studies using quantitative PCR in case/adjacent normal group and case/healthy normal group, so we couldn’t carry out the heterogeneity analysis. In this meta-analysis, although regular PCR was more frequently used for the detection of 4,977 bp deletion compared with quantitative PCR, quantitative PCR was more convenient and less time consume.

Therefore, the findings of mtDNA common deletion frequency in different cancers in this meta-analysis still require further replication with more precise analysis and larger studies to avoid the drawbacks.

Some limitations of the current study should be addressed. First, the total number of studies was too small to perform further subgroup analyses. Second, in the subgroup analysis by ethnicity, the included studies involved only EA and Asians, data concerning other ethnic groups such as Africans were not found. Third, only publications in English selected by databases were included in this meta-analysis. It is possible that some relevant publications or unpublished studies or publications in other languages were missed, which might place a bias on our results. Further, there is some evidence that age and cigarette smoke can alter the mtDNA 4,977 bp deletion frequency [47], [58]. However, due to the lack of original data in the publications, we didn’t stratify the studies by these factors. More accurate OR should be corrected by age, gender, smoking status, alcohol consumption and other exposure factors.

In conclusion, this study was the first meta-analysis to assess the association between mtDNA 4,977 bp deletion and cancers, and this paper provided evidence that mtDNA 4,977 bp deletion is more likely to happen in cancer patient, but is selected against in various types of cancerous tissues. However, due to the limitations of original studies included in the meta-analyses, further larger-sample studies using appropriate methods and subjects are required to evaluate the role of mtDNA 4,977 bp deletion in cancer development. In addition, such studies should also take environmental factors into account, so as to gain a better and more comprehensive understanding of unraveling the underlying mechanism of mtDNA 4,977 bp deletion in cancer development and progression.

## Supporting Information

Figure S1 **Sensitivity analysis of studies.** (A) case/adjacent normal group. (B) case/healthy normal group.
(TIF)

Figure S2 **Funnel plot analysis to detect publication bias.** (A) case/adjacent normal group. (B) case/healthy normal group.
(TIF)

## References

[1] Attardi G, Schatz G (1988) Biogenesis of mitochondria. Annu Rev Cell Biol 4: 289–333. 2461720 10.1146/annurev.cb.04.110188.001445

[2] Anderson S, Bankier AT, Barrell BG, de Bruijn MH, Coulson AR, et al (1981) Sequence and organization of the human mitochondrial genome. Nature 290: 457–465. 7219534 10.1038/290457a0

[3] Andrews RM, Kubacka I, Chinnery PF, Lightowlers RN, Turnbull DM, et al (1999) Reanalysis and revision of the Cambridge reference sequence for human mitochondrial DNA. Nat Genet 23: 147. 10508508 10.1038/13779

[4] Howell N, Kubacka I, Mackey DA (1996) How rapidly does the human mitochondrial genome evolve? Am J Hum Genet 59: 501–509. 8751850 PMC1914922

[5] Taylor RW, Turnbull DM (2005) Mitochondrial DNA mutations in human disease. Nat Rev Genet 6: 389–402. 15861210 10.1038/nrg1606 PMC1762815

[6] Warburg O (1956) On respiratory impairment in cancer cells. Science 124: 269–270. 13351639

[7] Brandon M, Baldi P, Wallace DC (2006) Mitochondrial mutations in cancer. Oncogene 25: 4647–4662. 16892079 10.1038/sj.onc.1209607

[8] Chatterjee A, Mambo E, Sidransky D (2006) Mitochondrial DNA mutations in human cancer. Oncogene 25: 4663–4674. 16892080 10.1038/sj.onc.1209604

[9] Czarnecka AM, Golik P, Bartnik E (2006) Mitochondrial DNA mutations in human neoplasia. J Appl Genet 47: 67–78. 16424612 10.1007/BF03194602

[10] Maitra A, Cohen Y, Gillespie SE, Mambo E, Fukushima N, et al (2004) The Human MitoChip: a high-throughput sequencing microarray for mitochondrial mutation detection. Genome Res 14: 812–819. 15123581 10.1101/gr.2228504 PMC479107

[11] Frenny VJ, Antonella Z, Luisa A, Shah AD, Sheth JJ, et al (2003) Cytogenetics and fluorescence in-situ hybridization in detection of hematological malignancies. Indian J Cancer 40: 135–139. 14716109

[12] Fliss MS, Usadel H, Caballero OL, Wu L, Buta MR, et al (2000) Facile detection of mitochondrial DNA mutations in tumors and bodily fluids. Science 287: 2017–2019. 10720328 10.1126/science.287.5460.2017

[13] Cortopassi GA, Arnheim N (1990) Detection of a specific mitochondrial DNA deletion in tissues of older humans. Nucleic Acids Res 18: 6927–6933. 2263455 10.1093/nar/18.23.6927 PMC332752

[14] Peng TI, Yu PR, Chen JY, Wang HL, Wu HY, et al (2006) Visualizing common deletion of mitochondrial DNA-augmented mitochondrial reactive oxygen species generation and apoptosis upon oxidative stress. Biochim Biophys Acta 1762: 241–255. 16368227 10.1016/j.bbadis.2005.10.008

[15] Holt IJ, Harding AE, Cooper JM, Schapira AH, Toscano A, et al (1989) Mitochondrial myopathies: clinical and biochemical features of 30 patients with major deletions of muscle mitochondrial DNA. Ann Neurol 26: 699–708. 2604380 10.1002/ana.410260603

[16] Corral-Debrinski M, Horton T, Lott MT, Shoffner JM, McKee AC, et al (1994) Marked changes in mitochondrial DNA deletion levels in Alzheimer brains. Genomics 23: 471–476. 7835898 10.1006/geno.1994.1525

[17] Berneburg M, Gattermann N, Stege H, Grewe M, Vogelsang K, et al (1997) Chronically ultraviolet-exposed human skin shows a higher mutation frequency of mitochondrial DNA as compared to unexposed skin and the hematopoietic system. Photochem Photobiol 66: 271–275. 9277148 10.1111/j.1751-1097.1997.tb08654.x

[18] Berneburg M, Grether-Beck S, Kurten V, Ruzicka T, Briviba K, et al (1999) Singlet oxygen mediates the UVA-induced generation of the photoaging-associated mitochondrial common deletion. J Biol Chem 274: 15345–15349. 10336420 10.1074/jbc.274.22.15345

[19] Wallace DC, Shoffner JM, Trounce I, Brown MD, Ballinger SW, et al (1995) Mitochondrial DNA mutations in human degenerative diseases and aging. Biochim Biophys Acta 1271: 141–151. 7599200 10.1016/0925-4439(95)00021-u

[20] Shen L, Fang H, Chen T, He J, Zhang M, et al (2010) Evaluating mitochondrial DNA in cancer occurrence and development. Ann N Y Acad Sci 1201: 26–33. 20649535 10.1111/j.1749-6632.2010.05635.x

[21] Meissner C, Bruse P, Mohamed SA, Schulz A, Warnk H, et al (2008) The 4977 bp deletion of mitochondrial DNA in human skeletal muscle, heart and different areas of the brain: a useful biomarker or more? Exp Gerontol 43: 645–652. 18439778 10.1016/j.exger.2008.03.004

[22] Lu J, Sharma LK, Bai Y (2009) Implications of mitochondrial DNA mutations and mitochondrial dysfunction in tumorigenesis. Cell Res 19: 802–815. 19532122 10.1038/cr.2009.69 PMC4710094

[23] Lee HC, Wei YH (2009) Mitochondrial DNA instability and metabolic shift in human cancers. Int J Mol Sci 10: 674–701. 19333428 10.3390/ijms10020674 PMC2660656

[24] Dani SU, Dani MA, Simpson AJ (2003) The common mitochondrial DNA deletion deltamtDNA(4977): shedding new light to the concept of a tumor suppressor mutation. Med Hypotheses 61: 60–63. 12781642 10.1016/s0306-9877(03)00105-1

[25] Aral C, Akkiprik M, Kaya H, Ataizi-Celikel C, Caglayan S, et al (2010) Mitochondrial DNA common deletion is not associated with thyroid, breast and colorectal tumors in Turkish patients. Genet Mol Biol 33: 1–4. 21637595 10.1590/S1415-47572009005000102 PMC3036096

[26] Lau J, Ioannidis JP, Schmid CH (1997) Quantitative synthesis in systematic reviews. Ann Intern Med 127: 820–826. 9382404 10.7326/0003-4819-127-9-199711010-00008

[27] Higgins JP, Thompson SG (2002) Quantifying heterogeneity in a meta-analysis. Stat Med 21: 1539–1558. 12111919 10.1002/sim.1186

[28] Mantel N, Haenszel W (1959) Statistical aspects of the analysis of data from retrospective studies of disease. J Natl Cancer Inst 22: 719–748. 13655060

[29] DerSimonian R, Laird N (1986) Meta-analysis in clinical trials. Control Clin Trials 7: 177–188. 3802833 10.1016/0197-2456(86)90046-2

[30] Egger M, Davey Smith G, Schneider M, Minder C (1997) Bias in meta-analysis detected by a simple, graphical test. BMJ 315: 629–634. 9310563 10.1136/bmj.315.7109.629 PMC2127453

[31] Zhu W, Qin W, Sauter ER (2004) Large-scale mitochondrial DNA deletion mutations and nuclear genome instability in human breast cancer. Cancer Detect Prev 28: 119–126. 15068836 10.1016/j.cdp.2003.11.008

[32] Pavicic WH, Richard SM (2009) Correlation analysis between mtDNA 4977-bp deletion and ageing. Mutat Res 670: 99–102. 19646455 10.1016/j.mrfmmm.2009.07.009

[33] Tseng LM, Yin PH, Tsai YF, Chi CW, Wu CW, et al (2009) Association between mitochondrial DNA 4,977 bp deletion and NAD(P)H:quinone oxidoreductase 1 C609T polymorphism in human breast tissues. Oncol Rep 21: 1169–1174. 19360290 10.3892/or_00000337

[34] Ye C, Shu XO, Wen W, Pierce L, Courtney R, et al (2008) Quantitative analysis of mitochondrial DNA 4977-bp deletion in sporadic breast cancer and benign breast diseases. Breast Cancer Res Treat 108: 427–434. 17541740 10.1007/s10549-007-9613-9 PMC3836503

[35] Tseng LM, Yin PH, Chi CW, Hsu CY, Wu CW, et al (2006) Mitochondrial DNA mutations and mitochondrial DNA depletion in breast cancer. Genes Chromosomes Cancer 45: 629–638. 16568452 10.1002/gcc.20326

[36] Tan DJ, Bai RK, Wong LJ (2002) Comprehensive scanning of somatic mitochondrial DNA mutations in breast cancer. Cancer Res 62: 972–976. 11861366

[37] Bianchi MS, Bianchi NO, Bailliet G (1995) Mitochondrial DNA mutations in normal and tumor tissues from breast cancer patients. Cytogenet Cell Genet 71: 99–103. 7606938 10.1159/000134072

[38] Dani MA, Dani SU, Lima SP, Martinez A, Rossi BM, et al (2004) Less DeltamtDNA4977 than normal in various types of tumors suggests that cancer cells are essentially free of this mutation. Genet Mol Res 3: 395–409. 15614730

[39] Gwak GY, Lee DH, Moon TG, Choi MS, Lee JH, et al (2011) The correlation of hepatitis B virus pre-S mutation with mitochondrial D-loop mutations and common deletions in hepatocellular carcinoma. Hepatogastroenterology 58: 522–528. 21661424

[40] Wheelhouse NM, Lai PB, Wigmore SJ, Ross JA, Harrison DJ (2005) Mitochondrial D-loop mutations and deletion profiles of cancerous and noncancerous liver tissue in hepatitis B virus-infected liver. Br J Cancer 92: 1268–1272. 15785740 10.1038/sj.bjc.6602496 PMC2361973

[41] Fukushima S, Honda K, Awane M, Yamamoto E, Takeda R, et al (1995) The frequency of 4977 base pair deletion of mitochondrial DNA in various types of liver disease and in normal liver. Hepatology 21: 1547–1551. 7768499

[42] Yin PH, Lee HC, Chau GY, Wu YT, Li SH, et al (2004) Alteration of the copy number and deletion of mitochondrial DNA in human hepatocellular carcinoma. Br J Cancer 90: 2390–2396. 15150555 10.1038/sj.bjc.6601838 PMC2409531

[43] Shao JY, Gao HY, Li YH, Zhang Y, Lu YY, et al (2004) Quantitative detection of common deletion of mitochondrial DNA in hepatocellular carcinoma and hepatocellular nodular hyperplasia. World J Gastroenterol 10: 1560–1564. 15162525 10.3748/wjg.v10.i11.1560 PMC4572754

[44] Wang J, Lu YY (2009) Mitochondrial DNA 4977-bp deletion correlated with reactive oxygen species production and manganese superoxidedismutase expression in gastric tumor cells. Chin Med J (Engl) 122: 431–436. 19302750

[45] Wu CW, Yin PH, Hung WY, Li AF, Li SH, et al (2005) Mitochondrial DNA mutations and mitochondrial DNA depletion in gastric cancer. Genes Chromosomes Cancer 44: 19–28. 15892105 10.1002/gcc.20213

[46] Kamalidehghan B, Houshmand M, Ismail P, Panahi MS, Akbari MH (2006) Delta mtDNA4977 is more common in non-tumoral cells from gastric cancer sample. Arch Med Res 37: 730–735. 16824932 10.1016/j.arcmed.2006.02.005

[47] Chen T, He J, Shen L, Fang H, Nie H, et al (2011) The mitochondrial DNA 4,977-bp deletion and its implication in copy number alteration in colorectal cancer. BMC Med Genet 12: 8. 21232124 10.1186/1471-2350-12-8 PMC3025938

[48] Upadhyay R, Jain M, Kumar S, ChandGhoshal U, Mittal B (2009) Role of mitochondrial DNA 4977-bp deletions in esophageal cancer susceptibility and prognosis in a northern Indian population. Cancer Genet Cytogenet 195: 175–178. 19963120 10.1016/j.cancergencyto.2009.06.017

[49] Abnet CC, Huppi K, Carrera A, Armistead D, McKenney K, et al (2004) Control region mutations and the ‘common deletion’ are frequent in the mitochondrial DNA of patients with esophageal squamous cell carcinoma. BMC Cancer 4: 30. 15230979 10.1186/1471-2407-4-30 PMC459226

[50] Tan BH, Skipworth RJ, Stephens NA, Wheelhouse NM, Gilmour H, et al (2009) Frequency of the mitochondrial DNA 4977 bp deletion in oesophageal mucosa during the progression of Barrett’s oesophagus. Eur J Cancer 45: 736–740. 19211242 10.1016/j.ejca.2009.01.013

[51] Lee HC, Yin PH, Yu TN, Chang YD, Hsu WC, et al (2001) Accumulation of mitochondrial DNA deletions in human oral tissues – effects of betel quid chewing and oral cancer. Mutat Res 493: 67–74. 11516716 10.1016/s1383-5718(01)00160-7

[52] Rahul Pandey DM CC, Vimal Choubey, Rajiv Sarin, Abbas Ali Mahdi, et al (2012) Association of mitochondrial deoxyribonucleic acid mutation with polymorphism in CYP2E1 gene in oral carcinogenesis. Journal of Oral Biology and Craniofacial Research 2: 4–9. 25756024 10.1016/S2212-4268(12)60003-7 PMC3941659

[53] Tan DJ CJ, Chen WL, Agress LJ, Yeh KT, et al (2003) Novel heteroplasmic frameshift and missense somatic mitochondrial DNA mutations in oral cancer of betel quid chewers. Genes Chromosomes Cancer 37: 186–194. 12696067 10.1002/gcc.10217

[54] Maximo V SP, Lima J, Cameselle-Teijeiro J, Sobrinho-Simoes M (2002) Mitochondrial DNA somatic mutations (point mutations and large deletions) and mitochondrial DNA variants in human thyroid pathology: a study with emphasis on Hurthle cell tumors. Am J Pathol 160: 1857–1865. 12000737 10.1016/S0002-9440(10)61132-7 PMC1850872

[55] Yang JH, Lee HC, Chung JG, Wei YH (2004) Mitochondrial DNA mutations in light-associated skin tumors. Anticancer Res 24: 1753–1758. 15274351

[56] Kamenisch Y, Wenz J, Metzler G, Bauer J, Neubauer H, et al (2007) The mitochondrial DNA common deletion is present in most basal and squamous cell carcinoma samples isolated by laser capture microdissection but generally at reduced rather than increased levels. J Invest Dermatol 127: 486–490. 17039246 10.1038/sj.jid.5700552

[57] Futyma K, Putowski L, Cybulski M, Miotla P, Rechberger T, et al (2008) The prevalence of mtDNA4977 deletion in primary human endometrial carcinomas and matched control samples. Oncol Rep 20: 683–688. 18695924

[58] Dai JG, Xiao YB, Min JX, Zhang GQ, Yao K, et al (2006) Mitochondrial DNA 4977 BP deletion mutations in lung carcinoma. Indian J Cancer 43: 20–25. 16763358 10.4103/0019-509x.25771

[59] Lewis PD, Baxter P, Paul Griffiths A, Parry JM, Skibinski DO (2000) Detection of damage to the mitochondrial genome in the oncocytic cells of Warthin’s tumour. J Pathol 191: 274–281. 10878549 10.1002/1096-9896(2000)9999:9999<::AID-PATH634>3.0.CO;2-U

[60] Kara M TA, Borekci B, Dagli F, Oztas S (2012) Mitochondrial DNA 4977 bp deletion in chronic cervicitis and cervix cancers. Balkan Journal of Medical Genetics 15: 25–29. 24052719 10.2478/v10034-012-0004-0 PMC3776654

[61] Wen Q, Hu Y, Ji F, Qian G (2011) Mitochondrial DNA alterations of peripheral lymphocytes in acute lymphoblastic leukemia patients undergoing total body irradiation therapy. Radiat Oncol 6: 133. 21978541 10.1186/1748-717X-6-133 PMC3198693

[62] Yu JJ, Yan T (2010) Effect of mtDNA mutation on tumor malignant degree in patients with prostate cancer. Aging Male 13: 159–165. 20136572 10.3109/13685530903536668

[63] Tan DJ, Chang J, Liu LL, Bai RK, Wang YF, et al (2006) Significance of somatic mutations and content alteration of mitochondrial DNA in esophageal cancer. BMC Cancer 6: 93. 16620376 10.1186/1471-2407-6-93 PMC1459869

[64] Rogounovitch TI, Saenko VA, Shimizu-Yoshida Y, Abrosimov AY, Lushnikov EF, et al (2002) Large deletions in mitochondrial DNA in radiation-associated human thyroid tumors. Cancer Res 62: 7031–7041. 12460924

[65] Kao S, Chao HT, Wei YH (1995) Mitochondrial deoxyribonucleic acid 4977-bp deletion is associated with diminished fertility and motility of human sperm. Biol Reprod 52: 729–736. 7779994 10.1095/biolreprod52.4.729

[66] Shenkar R, Navidi W, Tavare S, Dang MH, Chomyn A, et al (1996) The mutation rate of the human mtDNA deletion mtDNA4977. Am J Hum Genet 59: 772–780. 8808591 PMC1914802

[67] Thayer RE, Wittock R, Parr R, Zullo S, Birch-Machin MA (2003) A maternal line study investigating the 4977-bp mitochondrial DNA deletion. Exp Gerontol 38: 567–571. 12742534 10.1016/s0531-5565(03)00033-0

[68] Wang E, Wong A, Cortopassi G (1997) The rate of mitochondrial mutagenesis is faster in mice than humans. Mutat Res 377: 157–166. 9247611 10.1016/s0027-5107(97)00091-2

[69] Lee HC, Hsu LS, Yin PH, Lee LM, Chi CW (2007) Heteroplasmic mutation of mitochondrial DNA D-loop and 4977-bp deletion in human cancer cells during mitochondrial DNA depletion. Mitochondrion 7: 157–163. 17280876 10.1016/j.mito.2006.11.016

[70] Park JS, Sharma LK, Li H, Xiang R, Holstein D, et al (2009) A heteroplasmic, not homoplasmic, mitochondrial DNA mutation promotes tumorigenesis via alteration in reactive oxygen species generation and apoptosis. Hum Mol Genet 18: 1578–1589. 19208652 10.1093/hmg/ddp069 PMC2733816

