# Supplementary figures and images for: Mitochondrial Common Deletion, a Potential Biomarker for Cancer Occurrence, Is Selected against in Cancer Background: A Meta-Analysis of 38 Studies
Source: PLoS One. 2013 Jul 4;8(7):e67953. doi: 10.1371/journal.pone.0067953 (PMC3701633; doi:10.1371/journal.pone.0067953)

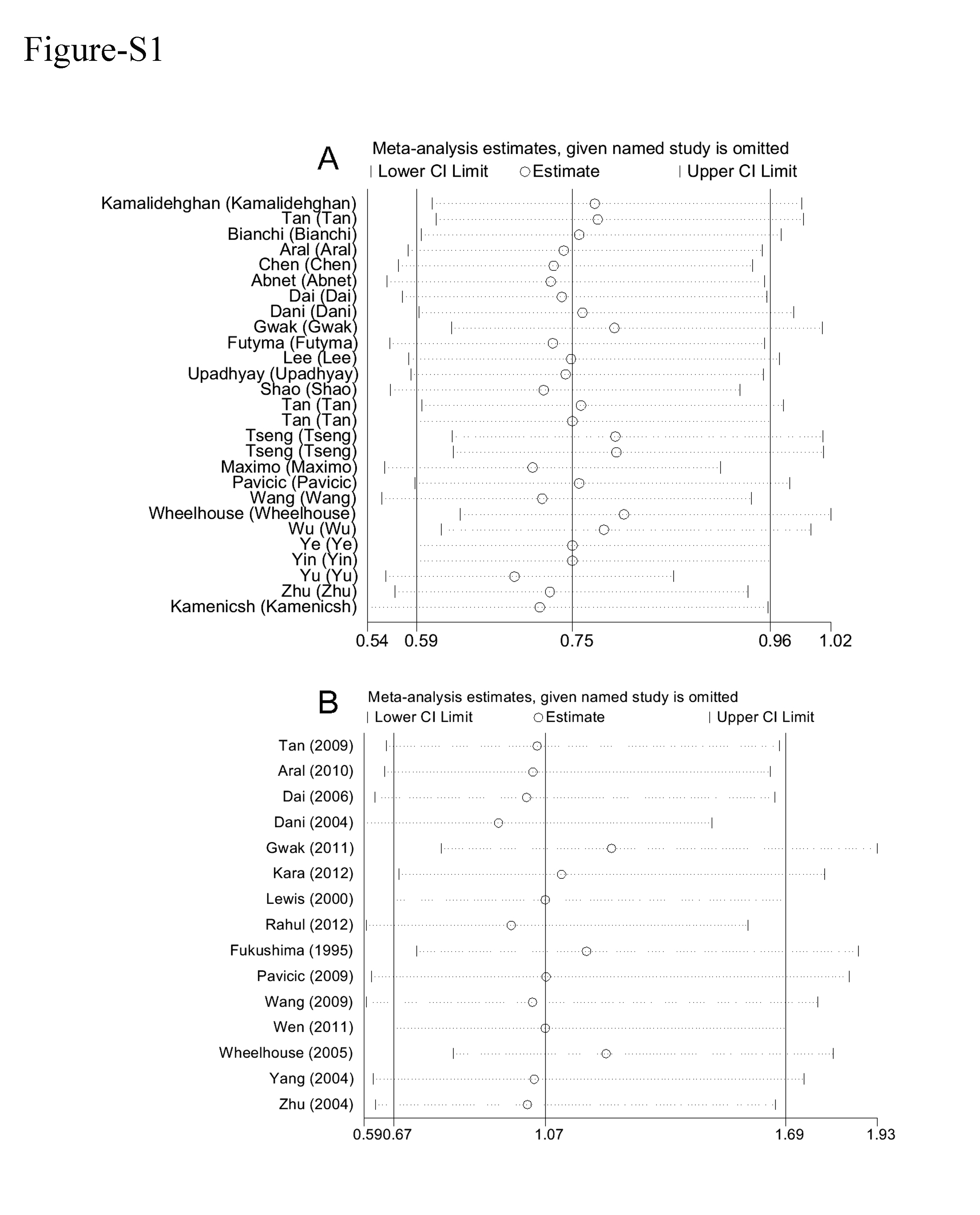

Supplement: Figure S1 — Sensitivity analysis of studies. (A) case/adjacent normal group. (B) case/healthy normal group. (TIF) [file pone.0067953.s001.tif]

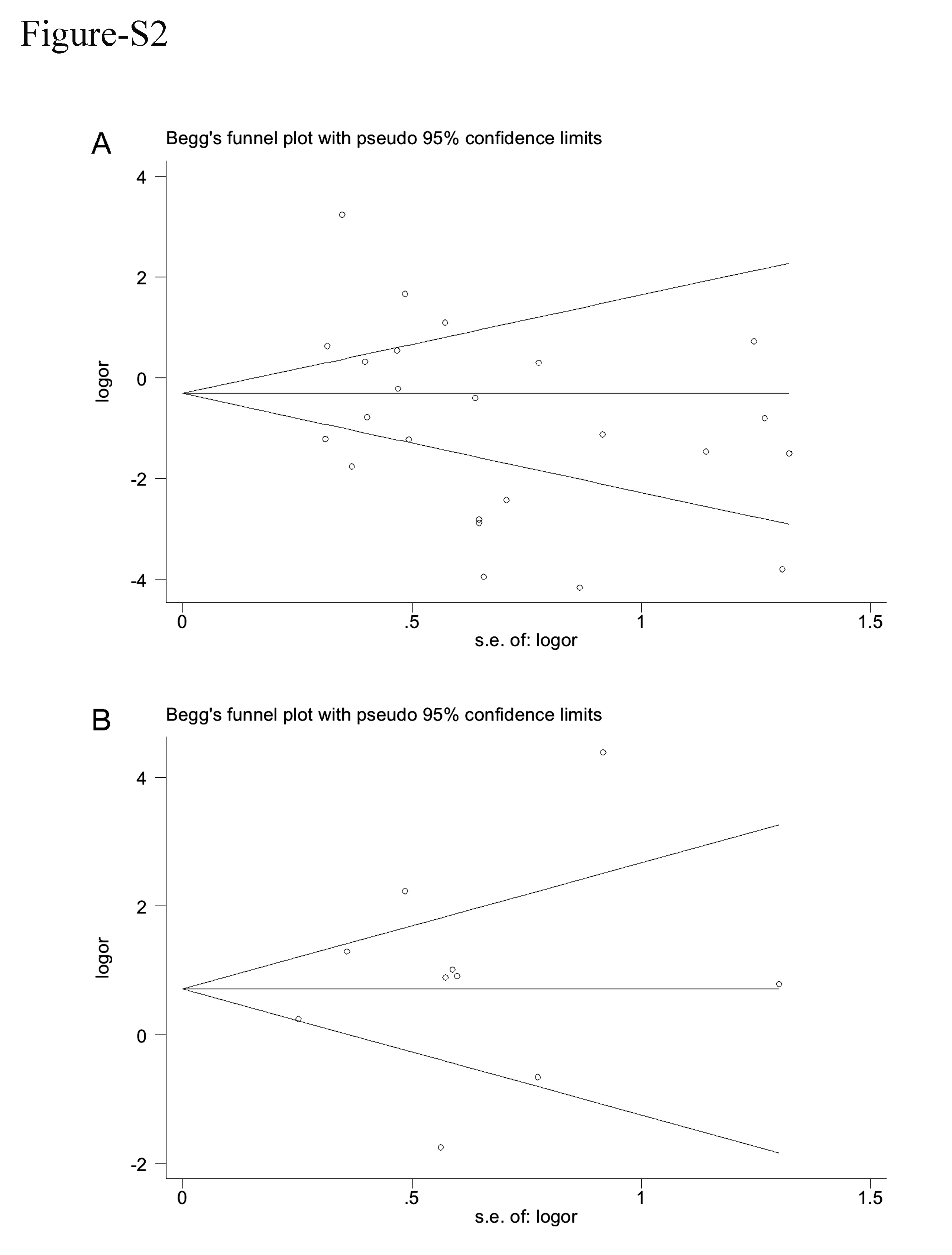

Supplement: Figure S2 — Funnel plot analysis to detect publication bias. (A) case/adjacent normal group. (B) case/healthy normal group. (TIF) [file pone.0067953.s002.tif]
